# Alveolar Abscess

**Published:** 1857-07

**Authors:** 


### EDITORIAL DEPARTMENT.

*Alveolar Abscess.*—Some twelve or fifteen years ago the senior editor published an account of alveolar abscess occasioned by the death of the upper central incisors—the matter from which was discharged behind the curtain of the palate into the fauces. He has subsequently met with two other cases of the same kind—one about two years ago and the other more recently. In the first, an abscess had formed in each of the sockets of the four incisors of the upper jaw. The subject of this case was a gentleman about twenty-eight years of age. A day or two before he called to consult the writer, the teeth being sore to the touch and the crowns very much decayed and partly broken off, he had had them extracted, consequently the escape of matter from behind the velum had nearly ceased. It had, however, been a source of no little annoyance and uneasiness to him, as, according to his own statement, it had been kept up, at times, for several months—the place of the formation of which not having been suspected either by himself or those whom he had consulted with a view of obtaining relief from this most annoying and singu lar affection. The writer, however, having had a similar case some years before, was enabled to detect the seat of the disease at once, and to relieve the apprehensions of the patient, by assuring him that, the cause being already removed, he would be completely restored in two or three days.

In this, as in the first case which came under the observation of the writer, the matter had evidently made for itself a passage through the nasal plates of the superior maxillary and from thence proceeded backward to the posterior nares, where perforating the mucous membrane, discharged itself behind the velum palati into the fauces. If it had escaped through the sockets of the teeth posteriorly, and from thence passed between the mucous membrane of the roof of the mouth and bones of the palate or the velum and then made an opening through the posterior part of this movable curtain for its exit, the route which the matter would have taken would have been indicated by heightened redness of the submucous tissue, but as no such indication was perceptible, the inference seems conclusive, that it traversed the upper surface of the nasal plates of the superior maxillary and palatine bones.

The subject of the last case was a maiden lady, about fifty years of age. The abscess was seated in the socket of the first left superior molar, at the extremity of the palatine root. The matter, apparently, had perforated directly through the bone of the palate, thence proceeded backward, and escaped in the manner as described in the first case. The abscess and discharge had continued several months, when the writer first saw the case, and besides the annoyance which it occasioned her general health had suffered considerably from it. Having pointed out the seat of the disease, and advised the extraction of the tooth as constituting the proper and only remedial indication, she at once submitted to the operation. Three days after the discharge from behind the velum had entirely ceased.

